# ResDAC-Net: a novel pancreas segmentation model utilizing residual double asymmetric spatial kernels

**DOI:** 10.1007/s11517-024-03052-9

**Published:** 2024-03-08

**Authors:** Zhanlin Ji, Jianuo Liu, Juncheng Mu, Haiyang Zhang, Chenxu Dai, Na Yuan, Ivan Ganchev

**Affiliations:** 1 Department of Artificial Intelligence, North China University of Science and Technology, Tangshan, 063009 China; 2 Department of Computing, Xi’an Jiaotong-Liverpool University, Suzhou, China; 3 Intelligence and Information Engineering College, Tangshan University, Tangshan, 063000 China; 4 Telecommunications Research Centre (TRC), University of Limerick, Limerick, V94 T9PX Ireland; 5 Department of Computer Systems, University of Plovdiv “Paisii Hilendarski”, Plovdiv, 4000 Bulgaria; 6 Institute of Mathematics and Informatics—Bulgarian Academy of Sciences, Sofia, 1040 Bulgaria

**Keywords:** ResDAC-Net, Pancreatic segmentation, Image segmentation, Medical image processing

## Abstract

The pancreas not only is situated in a complex abdominal background but is also surrounded by other abdominal organs and adipose tissue, resulting in blurred organ boundaries. Accurate segmentation of pancreatic tissue is crucial for computer-aided diagnosis systems, as it can be used for surgical planning, navigation, and assessment of organs. In the light of this, the current paper proposes a novel Residual Double Asymmetric Convolution Network (ResDAC-Net) model. Firstly, newly designed ResDAC blocks are used to highlight pancreatic features. Secondly, the feature fusion between adjacent encoding layers fully utilizes the low-level and deep-level features extracted by the ResDAC blocks. Finally, parallel dilated convolutions are employed to increase the receptive field to capture multiscale spatial information. ResDAC-Net is highly compatible to the existing state-of-the-art models, according to three (out of four) evaluation metrics, including the two main ones used for segmentation performance evaluation (i.e., DSC and Jaccard index).

**Graphical abstract:**

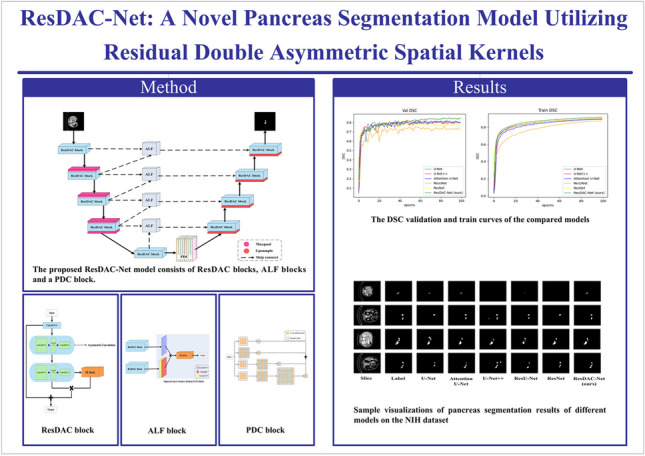

## Introduction

Pancreatic cancer is a highly malignant disease that is difficult to diagnose early and progresses rapidly [1–3]. Therefore, accurate pancreatic segmentation can effectively improve the diagnostic accuracy of pancreatic cancer and help doctors assess treatment progress. Doctors need to meticulously delineate patients’ medical images, converting 2D slices into 3D volumetric images in their minds [4]. This process allows them to understand the spatial relationships between pathological tissues and surrounding organs. It is essential for doctors to possess substantial experience in image interpretation. However, the presence of imaging artifacts, such as pseudo-shadows and gas artifacts, introduces noise interference that can potentially impact doctors’ assessments [5–7]. Additionally, different doctors may yield subjective and inconsistent diagnostic results, prone to misjudgment due to fatigue or insufficient experience. On the other hand, automatic pancreatic segmentation is a challenging task due to several reasons [8]. Firstly, the pancreas has a relatively small volume in the abdomen, requiring a considerable amount of non-target image information for organ segmentation [9]. Secondly, the shape and boundaries of the pancreas in computed tomography (CT) images are highly irregular and variable, even within adjacent 3D slices, exhibiting significant diversity [10]. Lastly, the pancreas exhibits similar intensity to surrounding organs, making it difficult to distinguish it using image enhancement techniques such as contrast, leading to unclear slice boundaries [11]. The application of deep learning techniques has shown promising results in addressing these pancreatic segmentation problems by offering a potential solution for quick and accurate extraction of pancreas regions in medical images [12, 13].

In recent years, 2D deep learning has been widely applied to pancreatic segmentation. These methods split the 3D volume data of CT images into 2D slices and perform segmentation on individual slices through pixel-wise classification. The U-Net model, proposed by Ronneberger et al. [14], is widely used for pixel-level classification tasks in medical images. However, the labeling of medical image datasets requires annotations from medical experts, resulting in a scarcity of clinically meaningful labeled datasets [15]. The U-Net model reduces the number of parameters by dynamically adjusting the number of channels and model depth for each layer [16]. Therefore, information from each scale of the image is meaningful [17]. The skip connections in the U-Net model address the issue of high-resolution feature loss during the up-sampling process, thereby enhancing the expression capability of local and global features [18, 19]. Expanding on this groundwork, researchers have put forth several enhancement strategies that integrate pancreatic features, aiming to further augment the effectiveness of segmentation [20].

Oktay et al. [21] integrated attention gates into the U-Net model, proposing the Attention U-Net architecture. The attention gates can suppress irrelevant parts of the model while enhancing the learning of task-relevant features [22]. This approach abandons the coarse-to-fine segmentation framework, which performed well in pancreatic segmentation, and eliminates the need for cascaded networks to achieve coarse segmentation and localization of organs. As a result, this approach performs well on various datasets. Cai et al. [23] utilized Convolutional Long Short-Term Memory (CLSTM) modules to address the problem of learning temporal features in pancreatic CT slice data. The CLSTM module achieves spatially consistent segmentation of individual slices for label predictions and further optimizes Convolutional Neural Network (CNN)-based segmentation results by considering the context features of the pancreas [24]. Heinrich et al. [25] proposed a high-performance ternary network architecture aiming to reduce reliance on computer memory [22].

In the current paper, we propose a pancreatic segmentation model, called ResDAC-Net, which utilizes a combination of residual networks and asymmetric convolutions to construct its backbone. The specific contributions of our work are as follows:

1. Based on the U-Net architecture, we replace two standard 3 × 3 convolutional blocks with newly designed Residual Double Asymmetric Convolution (ResDAC) blocks, which contain residual networks and asymmetric convolutions. This substitution enhances feature fitting, preserves more feature information in the target region, and alleviates issues like vanishing and exploding gradients.
2. We strengthen feature fusion between adjacent encoding layers through additional skip connections, thus fully utilizing the low-level and deep-level features extracted by the ResDAC blocks.
3. We employ parallel dilated convolutions with different dilation rates in a novel feature fusion PDC module, to increase the receptive field between encoding and decoding layers.
4. Inspired by ExFuse [26], we propose a new Adjacent Layer Feature fusion (ALF) block that performs feature fusion on additional skip connections between adjacent layers.

## Related work

### ResNet

CNNs are a popular topic in the field of image processing, particularly widely used in image segmentation. Among them, ResNet (Residual Network) is a special type of CNN architecture [27]. Compared to conventional CNN architectures, ResNet introduces residual units with identity mappings [28]. In traditional deep neural networks, as the number of layers increases, issues like vanishing or exploding gradients can occur, making it difficult for the network to converge [29, 30]. To address this problem, ResNet proposes the concept of residual units [31]. These units allow the network to directly learn the shallow representation of the data and then focus on learning the differences in deeper layers.

Specifically, ResNet achieves this by incorporating shortcut connections, also known as skip connections, which add the shallow input features to the deep output features. This enables the deep layers to directly reference information from the shallow layers, a connection referred to as “identity mapping” [32]. As a result, ResNet possesses enhanced learning capabilities and has demonstrated outstanding performance in various types of image segmentation tasks. Its essence lies in the fusion of two types of features from different receptive fields, using the fused features as the learning target, rather than simply relying on the output features from the previous layer [28].

### U-Net

U-Net is a deep learning network architecture used for image segmentation, particularly well-suited for biomedical image segmentation tasks. The design of U-Net draws inspiration from its U-shaped network structure, consisting of a down-sampling path (encoder) and an up-sampling path (decoder) [33]. The down-sampling path is similar to common CNNs. It gradually reduces the spatial dimensions of the image through a series of operations like convolution and pooling, while simultaneously extracting abstract features [34].

The up-sampling path in U-Net is different from traditional CNNs. In this path, U-Net employs operations like up-sampling to gradually restore low-resolution feature maps to prediction maps with the same size as the input image. Additionally, to fuse feature information from different scales, U-Net connects the features from the down-sampling path to the up-sampling path. This cross-layer connection helps the model better localize segmentation targets and preserve detailed information. The network architecture of U-Net allows it to perform effective image segmentation even with limited data, leading to remarkable success in the field of biomedical image segmentation.

### ACNet

ACNet is an improved CNN that enhances image recognition capabilities by introducing asymmetric convolutional kernels [35]. In traditional convolution operations, the kernels are symmetric. For example, a 3 × 3 convolutional kernel has the same weights in the horizontal and vertical directions. However, in real-world images, features in different directions may have varying importance. To better capture these features, the asymmetric convolutional network introduces asymmetric convolutional kernels, designed to have different weights in the horizontal and vertical directions. With this design, the network can better distinguish features in different directions within the image and more effectively capture directional information. Such asymmetric design enables the network to achieve better performance in image recognition tasks and provides new solutions for image segmentation tasks [36–38].

## Proposed model

### Residual double asymmetric convolution (ResDAC) block

In the usage of CNNs, when images are flipped or rotated, the image features extracted by standard square convolutions may change, leading to biases in the model’s segmentation results for the same object and reducing the model’s generalization ability. To address the issues associated with standard square convolution models, such as decreased accuracy due to parameter reduction, underfitting with separable convolutions, and model ineffectiveness at small input sizes, we propose a novel convolutional block, called ResDAC, based on spatial kernels. ResDAC aims to overcome the challenges posed by standard square convolutions by introducing a double asymmetric convolution approach. This new approach effectively addresses the decrease in accuracy caused by parameter reduction, mitigates underfitting issues with separable convolutions, and ensures the model’s effectiveness even at small input sizes.

ResDAC overcomes the problem of decreased accuracy when using standard square convolutions by combining residual networks with asymmetric convolutions. In this design, we connect two asymmetric convolutions using a residual network approach and incorporate the Squeeze-and-Excitation (SE) attention mechanism [39]. This allows us to capture channel dependencies after feature fusion, highlight important feature dimensions in the pancreas, and achieve superior accuracy while reducing the number of model’s parameters. This innovation liberates the model from the limitations of accuracy loss when dealing with standard square convolutions and effectively avoids overfitting and gradient explosion issues that may arise from deep network stacking. By introducing the ResDAC block, we aim to provide a more efficient and accurate solution, fully leveraging the advantages of spatial kernel-based convolution models and optimizing their performance across various tasks.

**Fig. 1:**
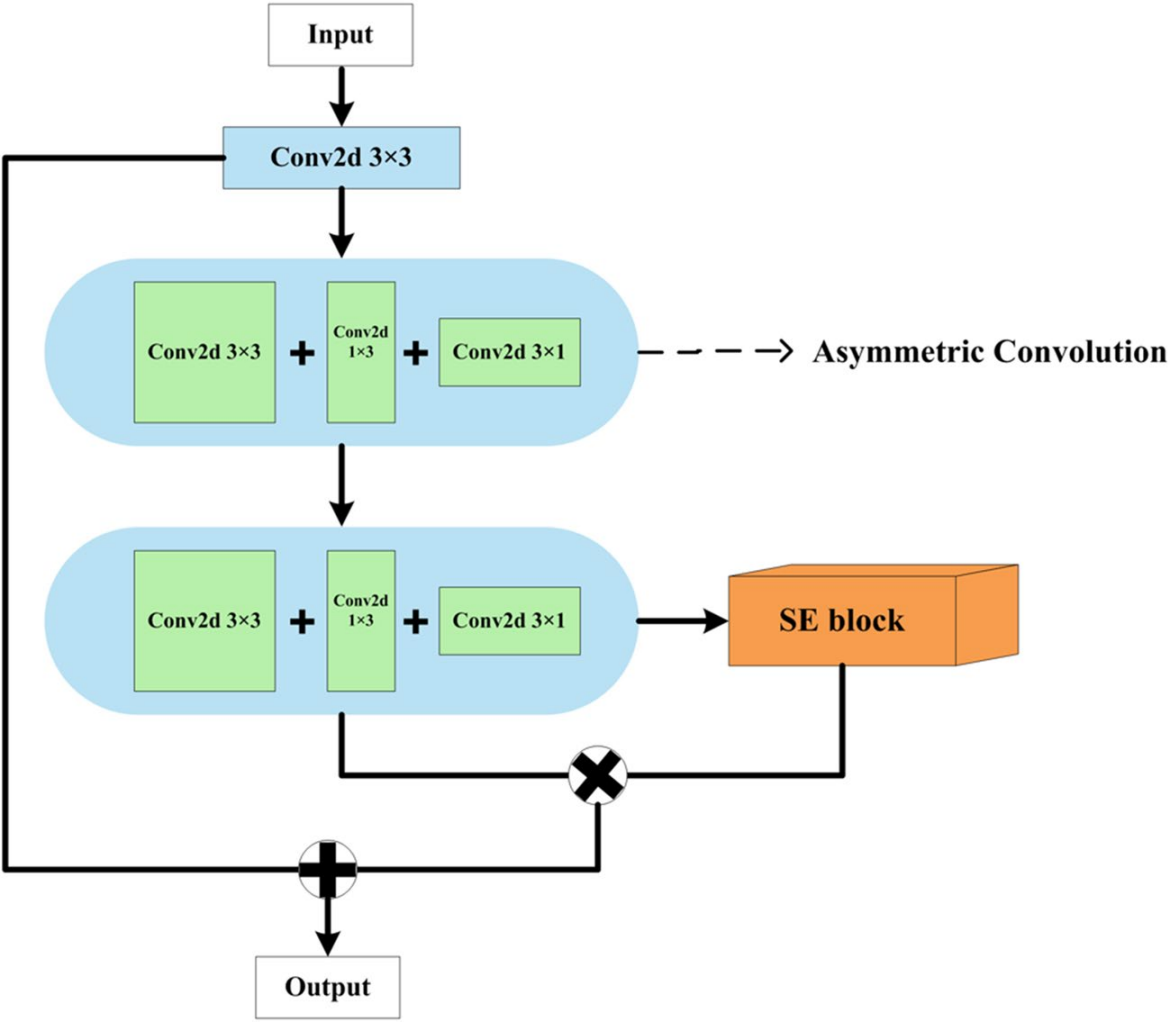
The elaborated ResDAC block

The elaborated ResDAC block takes input in the format of particular height ($H$) × width ($W$) × input channels $\left({C}_{in}\right)$ and outputs in the format of height ($H$) × width ($W$) × output channels $\left({C}_{out}\right)$. The block consists of one standard square convolutional layer (with a 3 × 3 kernel) and two asymmetric convolutional layers (with 3 × 3 kernels), uniquely forming a residual network convolutional block for discriminative feature extraction. In addition, the last layer also incorporates an SE block, as shown in Fig. 1.

Initially, the input feature map of size $H\times W\times {C}_{in}$ is fed into a 3 × 3 2D convolution. Let ${x}_{i}$ denote the input sample, where $i$ is the index of the convolutional layer. Then, the output after the convolution of the first layer is calculated similarly to [36], as follows:

1 $${P}_{i=1}= {[BNReLU\left({x}_{i=1}*{\omega }_{i=1}^{k\times k}+b\right)]}_{k=3}$$

where ${\omega }_{i=1}^{k\times k}$ denotes the kernel weights, $k$ denotes the size of the convolutional kernel, $b$ denotes the bias term, BNReLU refers to the combined operation of Batch Normalization (BN) and Rectified Linear Unit (ReLU) activation, symbol * denotes the convolution operation, and ${P}_{i=1}$ denotes the output feature map. In contrast to the initial layer, the second and third convolutional layers are composed of asymmetric convolutions with a kernel size of $k=3$. The convolutional output for these layers is calculated as follows:

2 $${P}_{i=2}= {[BNReLU\left({x}_{i=2}*{\omega }_{i=2}^{k\times k}+{x}_{i=2}*{\omega }_{i=2}^{1\times k}+{x}_{i=2}*{\omega }_{i=2}^{k\times 1}+b\right)]}_{k=3}$$

3 $${P}_{i=3}= {[BNReLU\left({x}_{i=3}*{\omega }_{i=3}^{k\times k}+{x}_{i=3}*{\omega }_{i=3}^{1\times k}+{x}_{i=3}*{\omega }_{i=3}^{k\times 1}+b\right)]}_{k=3}$$

The SE block performs the following operations:

4 $${A}_{sq}=\frac{1}{H\times W}{x}_{i=4}$$

5 $${A}_{ex}=\sigma [{W}_{2}*\left({W}_{1}*{A}_{sq}\right)]$$

6 $${P}_{i=4}={A}_{ex}\times {x}_{i=4}$$

where ${A}_{sq}$ denotes global average pooling on the input sample ${x}_{i=4}$ for the generation of a $1\times 1\times C$ feature vector; ${A}_{ex}$ is obtained through two fully connected layers (${W}_{1}$ and ${W}_{2}$), followed by a Sigmoid operation $\sigma$; and ${P}_{i=4}$ performs element-wise multiplication (denoted by ×) of the generated feature vector ${A}_{ex}$ and the weights from each of the $H\times W$ values in the input sample ${x}_{i=4}$ for each channel.

Then, the outputs of the last convolutional layer and the SE block are element-wise multiplied, and the result is element-wise added to the output of the first convolutional layer to strengthen feature extraction and obtain discriminative representations. So, the ResDAC block’s output ${P}_{out}$ is obtained as follows:

7 $${P}_{out}={P}_{i=3}\times {P}_{i=4}+{P}_{i=1}$$

By combining the outputs of multiple different convolutional layers through element-wise multiplication and addition, further feature fusion is achieved in generating the ResDAC output. The effectiveness of this asymmetric residual block lies in its ability to circumvent issues such as overfitting and gradient explosion, which may arise from the stacking of deep networks. Simultaneously, it attains superior performance.

### Adjacent Layer Feature fusion (ALF) block

**Fig. 2:**
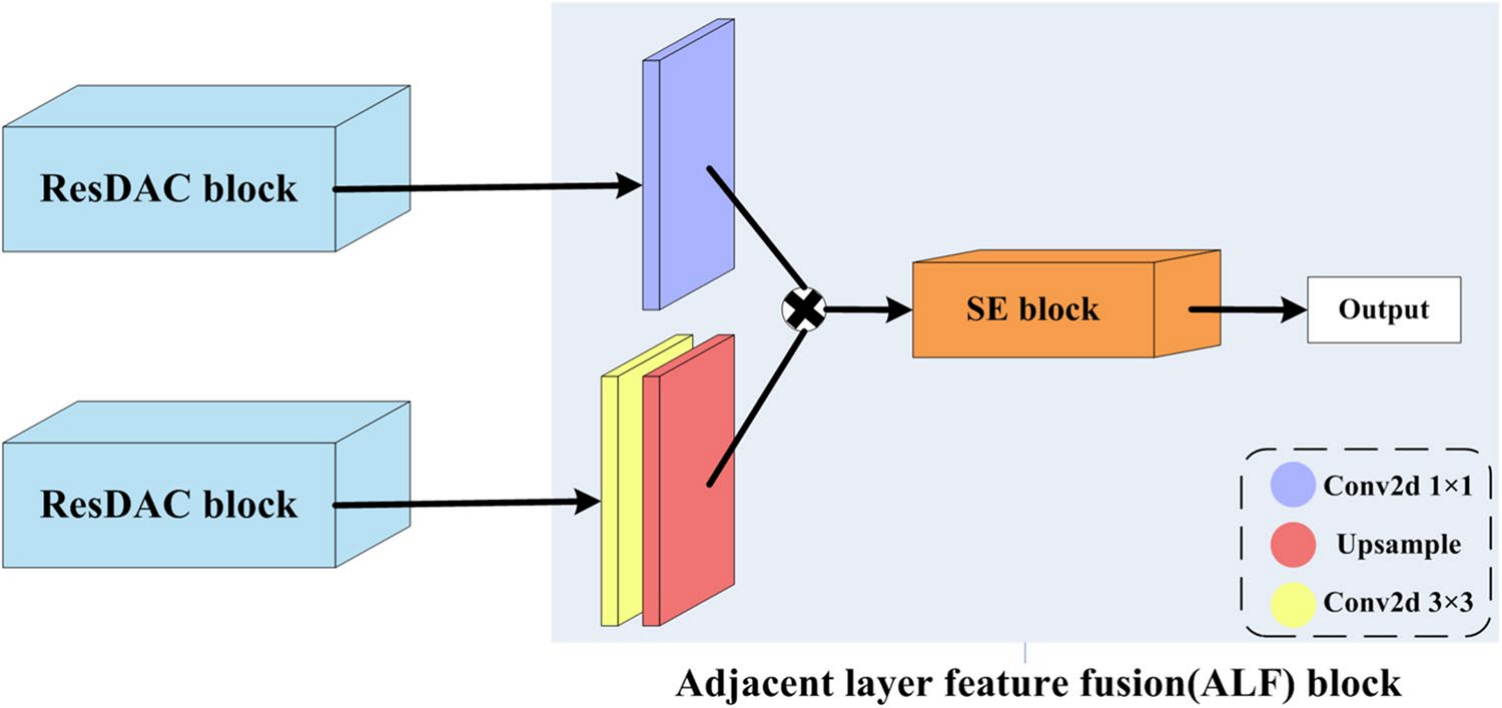
The elaborated ALF block

We have also designed a novel feature fusion block, called ALF, which fuses the outputs of adjacent convolutional blocks during the encoding stage to refine and enrich the feature maps. This block utilizes a 3 × 3 2D convolution to transform the high-level feature maps into feature maps at the same level as the previous layer, facilitating the extraction of detailed information from the high-level features and improving the capturing of pancreatic boundary features. For the low-level feature maps, a 1 × 1 2D convolution is applied to introduce non-linearity and expand the model’s options. This is why a “deep” network is preferable to a “wide” network [36], as the non-linear layer expands the model’s capacity. The two processed feature maps are then fused through element-wise multiplication, and an SE block is employed for implementing an attention mechanism as to explicitly model the interdependence between channels and adaptively recalibrate the channel-wise characteristics, thus enhancing the representation capability of the convolutional block for fused information. This improves boundary misalignment and forms an effective attention-based feature fusion execution framework. The structure of the elaborated ALF block is shown in Fig. 2.

### Parallel dilated convolutions (PDC) block

**Fig. 3:**
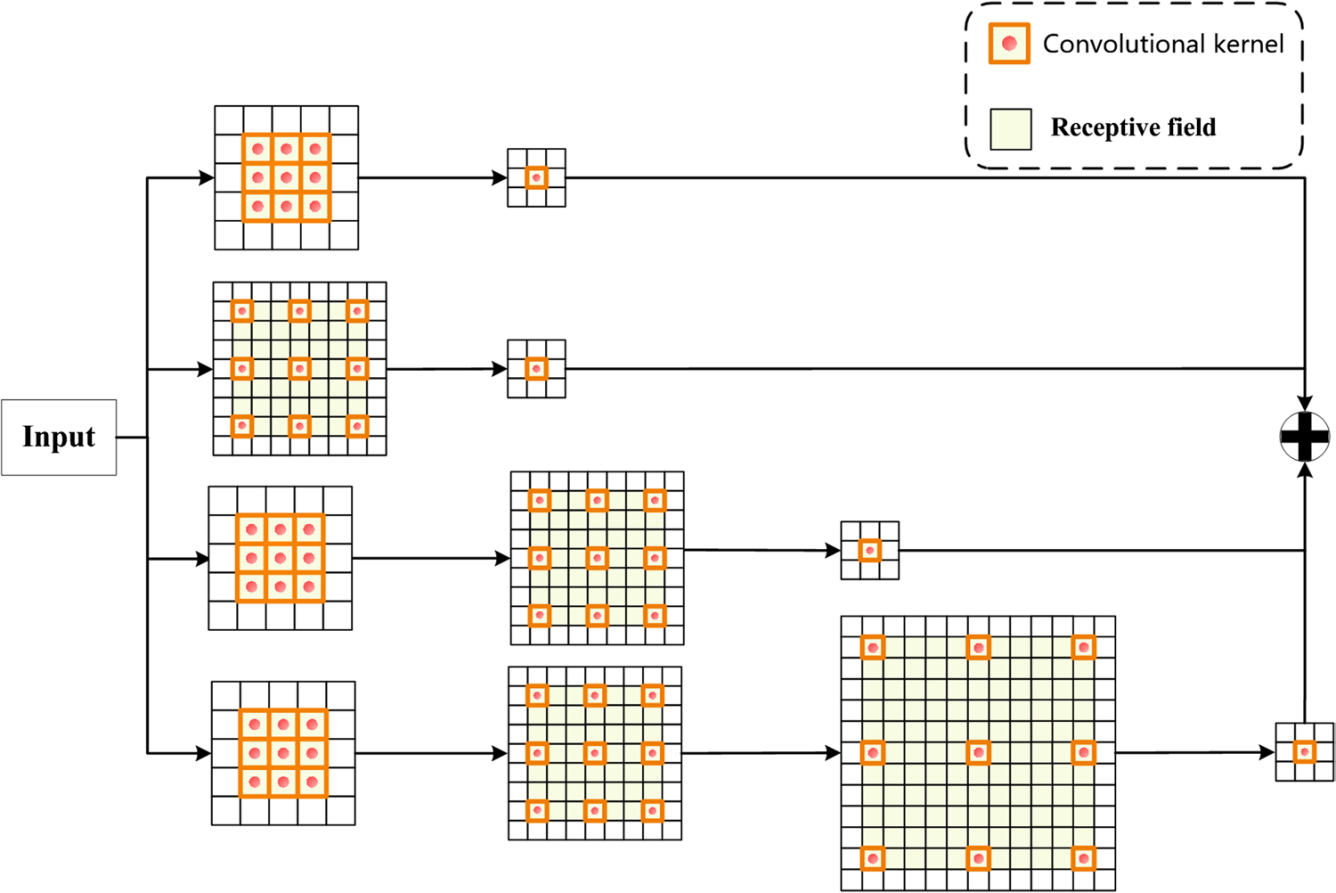
The elaborated PDC block

Compared to standard convolutions, dilated convolutions, also known as atrous convolutions, can enlarge the receptive field of the convolutional kernel by changing the dilation rate, thereby capturing multi-scale information from the feature maps. When the dilation rate is set to 1, the receptive field size of the dilated convolution is the same as that of the standard convolution. However, when the dilation rate is greater than 1, dilated convolutions can obtain a larger receptive field size and capture richer image information than standard convolutions [40]. In traditional convolution operations, a single convolutional kernel can only capture limited ranges of input features, thus restricting the network’s ability to understand large-scale information. Therefore, parallel dilated convolutions, through clever design, allow multiple convolutional kernels to parallelly span different receptive field sizes, providing a broader perception range. Parallel dilated convolutions introduce multiple parallel branches within the convolutional kernel, each with a different dilation rate, which allows to control the sampling span of the kernel when taking input. By using parallel convolutional kernels with different dilation rates, the network can capture multi-scale input features, including both local and global ones. Furthermore, compared to traditional dilated convolutions, parallel dilations can reduce the number of parameters and computational complexity of a model to some extent. This is because they share some convolutional layer parameters, and the computations of multiple branches can be executed in parallel. The calculation of the receptive field size for dilated convolutions is done according to the following formula:

8 $${M}_{i}={m}_{i}+\left({k}_{i}-1\right){\prod }_{i}^{n}{s}_{i}$$

where ${M}_{i}$ denotes the receptive field after dilation for the $i$-th layer, ${m}_{i}$ denotes the actual receptive field for the $i$-th layer, ${k}_{i}$ denotes the kernel size used in the dilated convolution for the $i$-th layer, ${s}_{i}$ denotes the offset of a convolutional kernel expanding outward, and ${\prod }_{i}^{n}{s}_{i}$ denotes the cumulative offset at which each convolutional kernel expands outward. The structure of the designed PDC block is illustrated in Fig. 3.

### ResDAC-Net

**Fig. 4:**
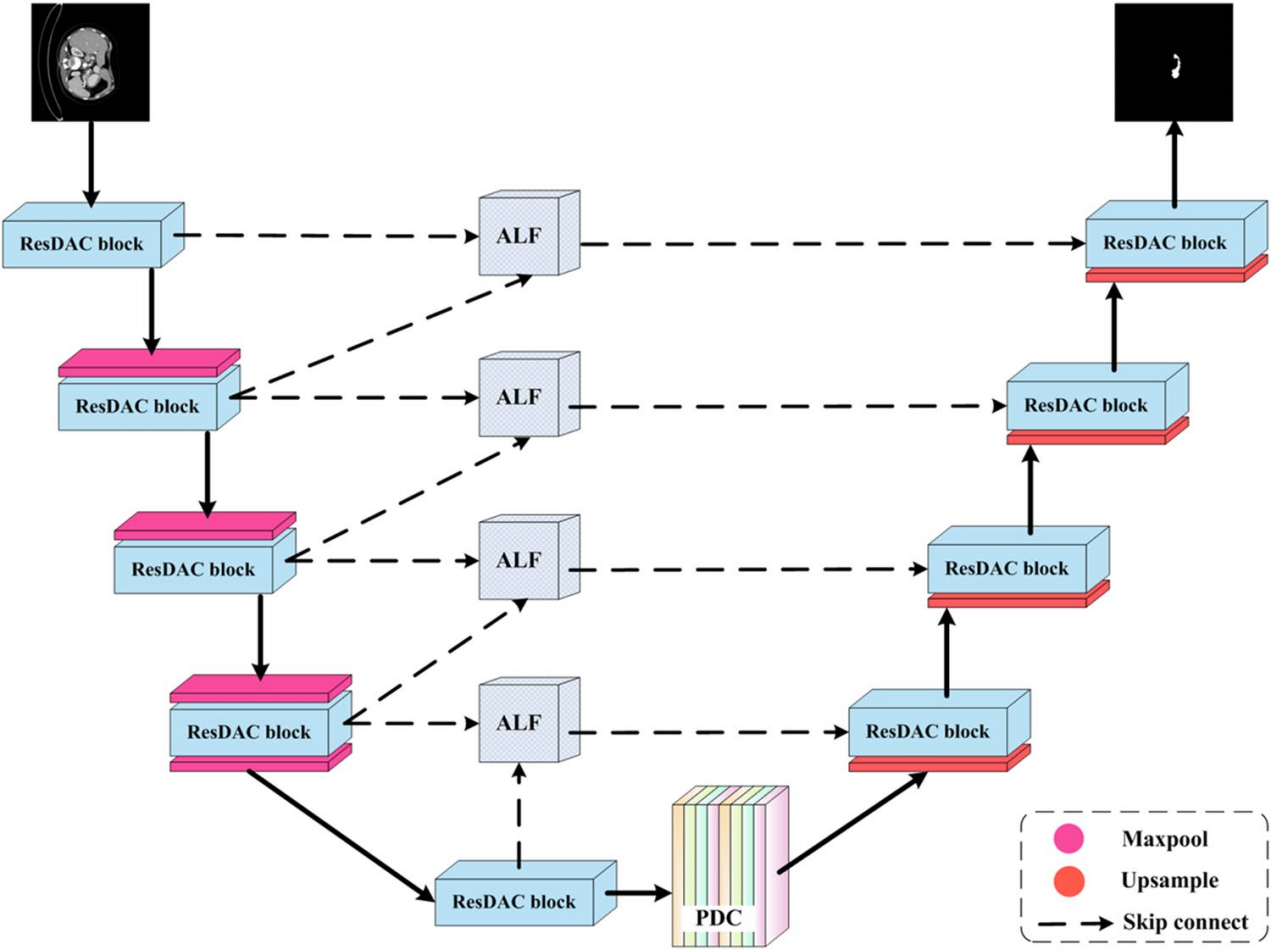
The proposed ResDAC-Net model

The proposed ResDAC-Net model consists of three core types of blocks, described in the previous subsections, as detailed in Fig. 4. The U-shaped encoder-decoder form of U-Net is adopted as a basic backbone. The encoder performs down-sampling on the input image and extracts essential features from the image. The encoder path is composed of a series of ResDAC blocks, followed by max-pooling. The output of a ResDAC block is combined with a 2 × 2 max-pooling to form an encoding unit (down-sampling unit) as follows:

9 $${E}_{j}=Maxpool\left({P}_{out},2\right)$$

where ${E}_{j}$ denotes the output of the $j$-th encoding layer, $Maxpool$ denotes the max-pooling operation, and ${P}_{out}$ denotes the output of the $j$-th ResDAC block.

Similarly to the encoder path, the decoder path is also composed of a series of ResDAC blocks for feature extraction. Additionally, before the skip connection, an up-sampling operation is applied to further refine the output features. The up-sampling unit with a scale of 2 is used to up-sample the feature map received from the lower layers of the network, as shown below:

10 $${up}_{j}=Upsample\left({x}_{j-1},2\right)$$

The up-sampled output, denoted as ${up}_{j}$, is further feature fused with the output of the ALF block at the corresponding encoder layer through a skip connection. By copying the feature maps from the encoder, the model can capture multi-scale information. The skip connections allow utilization of both high-resolution and low-resolution information from the feature maps during the up-sampling process [36], as follows:

11 $${D}_{j}={P}_{out}\left\{concat\left[{up}_{j},ALF\left({E}_{j},{E}_{j-1}\right)\right]\right\}$$

where ${D}_{j}$ denotes the output of the $j$-th decoder layer, $concat$ denotes the skip connection, $ALF\left({E}_{j},{E}_{j-1}\right)$ denotes the output of the feature fusion through the ALF block between the $j$-th and $(j-1)$-th encoder layers, and ${P}_{out}$ denotes the output after the convolution with the ResDAC block.

### Training and optimization methods

The proposed ResDAC-Net model utilizes a composite BCE-Dice loss and determines appropriate weight factors based on experimental results. The BCE-Dice loss [41] combines the binary cross-entropy (BCE) loss [42] and Dice loss [43], which are commonly used for binary segmentation tasks. The BCE loss is defined in [44], as follows:

12 $$BCE\left(p,q\right)=-\frac{1}{N}{\sum }_{i=1}^{N}\left({y}_{i}log\left({p}_{i}\right)+\left(1-{y}_{i}\right)log\left(1-{p}_{i}\right)\right)$$

where $N$ denotes the number of pixels, ${y}_{i}$ denotes the actual label (0 or 1) of the $i$-th pixel, and ${p}_{i}$ denotes the predicted probability of the $i$-th pixel belonging to a class [45].

The Dice loss is calculated as follows:

13 $$Dice(p,q)=\frac{2\sum_{i=1}^{N}{p}_{i}\cdot {q}_{i}}{\sum_{i=1}^{N}{p}_{i}^{2}+\sum_{i=1}^{N}{q}_{i}^{2}}$$

where ${q}_{i}$ denotes the target label of the $i$-th pixel, which is the binarized ground-truth label.

The composite BCE-Dice loss is calculated as follows:

14 $$BCEDice(p,q)=\alpha \cdot BCE(p,q)+(1-Dice(p,q))$$

where $\alpha$ denotes the weight factor.

As an optimizer, the Adam optimizer [46], which adapts the learning rate based on the first and second moment estimates of the gradients, was used, as it converges quickly during training and avoids getting stuck in local minima. In each iteration step $t$ ($t=\{1, 2,\dots ,T\}$), the following computations are performed:

15 $${m}_{t}={\beta }_{1}\cdot {m}_{t-1}+(1-{\beta }_{1})\cdot {g}_{t}$$

16 $${v}_{t}={\beta }_{2}\cdot {v}_{t-1}+(1-{\beta }_{2})\cdot {g}_{t}^{2}$$

where ${g}_{t}$ denotes the gradient at the current iteration step $t$, ${m}_{t}$ denotes the exponentially decaying average of the gradients, ${v}_{t}$ denotes the exponentially decaying average of the squared gradients (uncentered variance), and ${\beta }_{1}$ and ${\beta }_{2}$ denote the decay rates. Then, the bias-corrected estimates are calculated as follows:

17 $${\hat{m}}_{t}=\frac{{m}_{t}}{1-{\beta }_{1}^{t}}$$

18 $${\hat{m}}_{t}=\frac{{m}_{t}}{1-{\beta }_{1}^{t}}{\hat{v}}_{t}=\frac{{v}_{t}}{1-{\beta }_{2}^{t}}$$

The parameters are updated as follows:

19 $${\theta }_{t+1}={\theta }_{t}-\frac{\eta }{\sqrt{\hat{v}t}+\epsilon }\cdot {\hat{m}}_{t}$$

where $\eta$ denotes the learning rate, $\epsilon$ denotes a small constant used to prevent division by 0, and ${\theta }_{t+1}$ denotes the updated parameter.

The hardware configuration used in the conducted experiments, described in the next section, includes an Intel Core i5-12490 processor with a clock speed of 3.0 GHz, and a single NVIDIA RTX3060 graphics card with 12-GB memory. The neural model’s hyperparameters for training were set as follows: Batch_Size = 4, Epochs = 100 (validation was performed on each epoch, and the model was trained using the Adam optimizer), Initial_Learning_Rate = $1\times {10}^{-4}$, momentum = 0.9, Minimum_Learning_Rate = $1\times {10}^{-5}$. The network structure is implemented by using PyTorch.

## Experiments and results

### Datasets and evaluation metrics

CT image slices from the pancreas CT dataset of the National Institutes of Health (NIH) Clinical Center Cancer Imaging Archive [47–49] and the Medical Segmentation Decathlon (MSD) dataset [50] were used in the conducted pancreatic segmentation experiments, since these are mainstream datasets for pancreatic organ segmentation. 

The NIH dataset contains 82 abdominal contrast-enhanced CT scans. The ground-truth annotations for the pancreatic region in each CT scan were provided by experienced radiologists. The CT scan images are stored in the Digital Imaging and Communications in Medicine (DICOM) medical image format. Each CT scan has an original size of 512 × 512 pixels, and the number of slices varies between 181 and 466 for different patients. The slice thickness ranges from 1.5 to 2.5 mm. The scans were performed using Philips and Siemens multidetector computed tomography (MDCT) machines. For the experiments, only the CT scans, containing a pancreas image, were selected. To ensure a fair comparison, this dataset was divided into 60 individuals’ slices for the training set, whereas the validation and test sets contained slices from 11 individuals each.

The MSD dataset contains 281 abdominal contrast-enhanced CT scans. Each CT scan has an original size of 512 × 512 pixels. The slice thickness ranges from 0.7 to 7.5 mm. For the experiments, only the CT scans, containing a pancreas image, were selected. To ensure a fair comparison, this dataset was split in a 3:1:1 ratio for creating the training, testing, and validation sets.

In order to objectively evaluate the performance, training was conducted on the same dataset while keeping certain parameters constant. Common metrics such as Dice similarity coefficient (DSC), precision, recall, and Jaccard index were used to evaluate the results. These metrics are defined as follows:

20 $${\text{DSC}}=\frac{2TP}{2TP+FP+FN}$$

21 $${\text{Precision}}=\frac{TP}{TP+FP}$$

22 $${\text{Recall}}=\frac{TP}{TP+FN}$$

23 $$\mathrm{Jaccard\;index}=\frac{TP}{TP+FN+FP}$$

where $TP$ denotes the true positive counts, $FP$ denotes the false positive counts, and $FN$ denotes the false negative counts. These selected metrics provide a comprehensive evaluation of the segmentation results, enabling a fair comparison of different models on the same dataset.

### Ablation study experiments

To evaluate the effectiveness of different blocks incorporated into the baseline (U-Net), ablation study experiments were conducted on the NIH dataset first and then repeated on the MSD dataset. In these experiments, the elaborated ResDAC blocks, ALF blocks, and PDC block were subsequently added one by one to U-Net, by keeping the blocks added in the previous steps. The obtained results, presented in Tables 1 and 2, show gradual improvements in segmentation performance in each step, compared to the previous step, based on three (out of four) metrics, including the two main ones used for segmentation performance evaluation (i.e., DSC and Jaccard index). The only exception was precision, where the second step performed on the NIH dataset, resp. first step on MSD dataset, achieved a better result than the final step.

**Table 1:** Ablation study results on NIH dataset

Models	DSC (%)	Precision (%)	Recall (%)	Jaccard index (%)
U-Net (*baseline*)	78.69	77.88	80.88	65.66
U-Net + ResDAC	82.42	81.77	84.04	70.75
U-Net + ResDAC + ALF	83.89	**83.58**	85.10	72.74
**U-Net + ResDAC + ALF + PDC** **(i.e., ResDAC-Net)**	**84.56**	81.69	**88.48**	**73.70**

The best results are shown in bold

**Table 2:** Ablation study results on MSD dataset

Models	DSC (%)	Precision (%)	Recall (%)	Jaccard index (%)
U-Net (*baseline*)	64.36	69.26	66.78	53.40
U-Net + ResDAC	67.07	**71.68**	69.05	56.64
U-Net + ResDAC + ALF	68.21	69.41	71.66	57.77
**U-Net + ResDAC + ALF + PDC** **(i.e., ResDAC-Net)**	**69.15**	71.10	**72.31**	**58.76**

The best results are shown in bold

More specifically, in the first step performed on the NIH (resp. MSD) dataset, adding ResDAC blocks to the baseline model allowed to increase DSC by 3.73 (resp. 2.71) percentage points, precision by 3.89 (resp. 2.42) percentage points, recall by 3.16 (resp. 2.27) percentage points, and Jaccard index by 5.09 (resp. 3.24) percentage points. This indicates that the ResDAC blocks enable effective utilization of channel information from different receptive fields and adaptive refinement of encoded features to retain multi-scale information. Additionally, these blocks allowed the model to better distinguish features in different directions and capture specific directional information more effectively. In the second step, the addition of ALF blocks allowed to further improve the metrics values (except for precision when experimenting on the MSD dataset) by 1.47 (resp. 1.14) percentage points for DSC, 1.81 points for precision, 1.06 (resp. 2.61) percentage points for recall, and 1.99 (resp. 1.13) percentage points for Jaccard index. This highlights the explicit modeling of channel dependencies using the ALF blocks, enabling adaptive recalibration of channel characteristics, which enhances the convolutional block’s ability to represent fused information, improves mismatched boundaries, and provides powerful feature extraction capabilities for complex edges. Finally, in the last step, the addition of the PDC block allowed to further improve the metrics values (except for precision when experimenting on the NIH dataset) by 0.67 (resp. 0.94) percentage points for DSC, 1.69 percentage points for precision, 3.38 (resp. 0.65) percentage points for recall, and 0.96 (resp. 0.99) percentage points for Jaccard index. This indicates that using parallel dilated convolutions of different scales before the decoding stage allows to fuse feature maps with different scales and semantics, resulting in different scales of perception fields and effective extraction of multi-scale features.

Overall, the ablation study experiments demonstrated the effectiveness of the resultant ResDAC-Net structure in improving segmentation accuracy and effectively capturing multi-scale features in complex image segmentation tasks. Obviously, the combination of ResDAC, ALF, and PDC blocks plays a crucial role in achieving state-of-the-art segmentation performance.

### Performance comparison with U-Net–based models and ResNet models on NIH dataset

In the second set of experiments, the NIH dataset was used for pancreas segmentation performance comparison of the proposed ResDAC-Net model with U-Net–based models and ResNet models, namely U-Net [14], Attention U-Net [21], U-Net++ [51], ResUNet [52], and ResNet [27]. The obtained results are shown in Table 3.

**Table 3:** Pancreas segmentation performance comparison results obtained on NIH dataset

Models	DSC (%)	Precision (%)	Recall (%)	Jaccard index (%)
U-Net	78.69	77.88	80.88	65.66
Attention U-Net	79.77	80.64	80.60	67.25
U-Net++	79.41	73.49	88.07	66.62
ResUNet	77.09	81.37	74.87	63.79
ResNet	78.52	78.15	80.61	65.80
**ResDAC-Net (*ours*)**	**84.56**	**81.69**	**88.48**	**73.70**

The best results are shown in bold

As can be seen from Table 3, ResDAC-Net outperforms all other models, taking part in this comparison, based on all evaluation metrics. More specifically, the second-best performing model was left behind by 4.79 percentage points (w.r.t. Attention-UNet) according to DSC, 0.32 percentage points (w.r.t. ResU-Net) based on precision, 0.41 percentage points (w.r.t. UNet++) according to recall, and 6.45 percentage points (w.r.t. Attention-UNet) based on Jaccard index. These results demonstrate the effectiveness of ResDAC-Net in performing pancreas segmentation tasks, and its strong capabilities in extracting features from small objects and learning meaningful representations. This is due to the fact that ResDAC-Net takes into consideration the channel information, which allows it to extract the intra-slice relationships between pancreas and background pixels. Additionally, the inclusion of attention modules enables the effective utilization of multi-scale information among feature maps, facilitating communication and integration of features from different levels. As a result, ResDAC-Net can capture multi-scale targets, reduce the impact of inter-class imbalance, and adequately learn the relationship between the overall structure and fine details in pancreas images, leading to improved segmentation results.

**Fig. 5:**
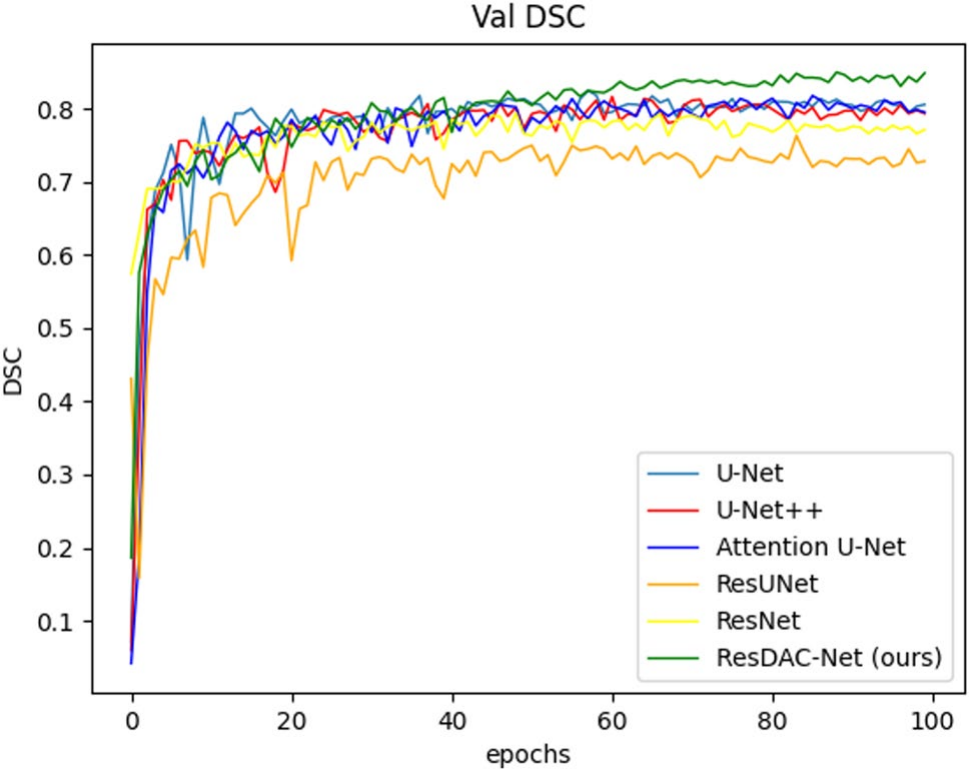
The DSC validation curves of the compared models

**Fig. 6:**
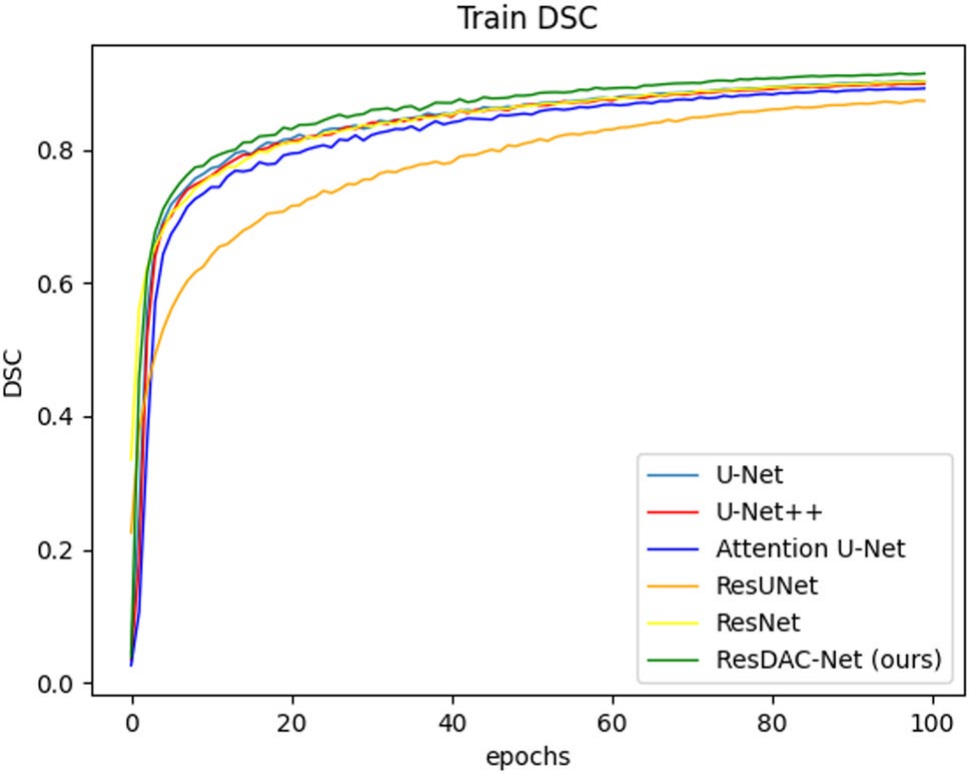
The DSC training curves of the compared models

The DSC validation and training curves of the compared models are shown in Figs. 5 and 6, respectively. From these figures, it is evident that the ResDAC-Net validation and training (represented by green color) converge faster than that of the other models.

**Fig. 7:**
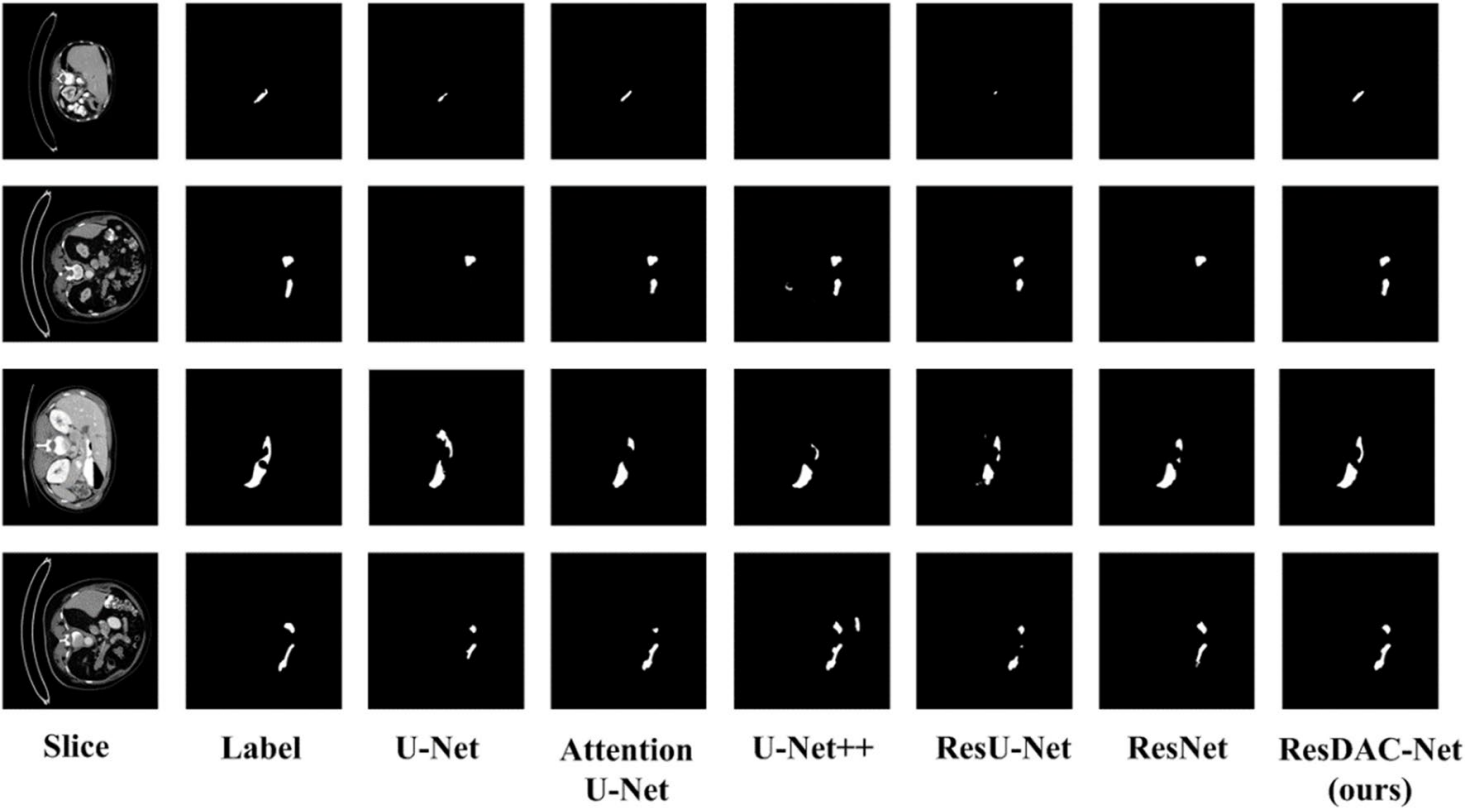
Sample visualizations of pancreas segmentation results of different models on NIH dataset

Figure 7 contains sample visualizations of pancreas segmentation results achieved by the compared models on the NIH dataset. The proposed ResDAC-Net model achieves more accurate pancreas segmentation compared to other models. It outperforms them in extracting pancreas edge pixels and effectively suppresses similar background information and noise surrounding the pancreas. Overall, ResDAC-Net is more effective in capturing detailed features of the pancreas, leading to improved segmentation performance.

### Performance comparison with U-Net–based models and ResNet models on MSD dataset

In order to validate the generalization ability of the proposed ResDAC-Net model, pancreas segmentation experiments were conducted with it, along with U-Net–based models and ResNet models, using a different dataset (i.e., MSD) from that on which the model was trained (i.e., NIH). The obtained results are shown in Table 4. As can be seen, ResDAC-Net achieved the highest value (among all compared models) for one of the main metrics used for segmentation performance evaluation, by scoring 69.15% for DSC. This result demonstrates robust generalization and strong pancreas pixel recognition capabilities of the proposed model across different datasets. On the other evaluation metrics, the proposed model took third place respectively on each one. While there were variations in the ResDAC-Net performance here, it is noteworthy that the baseline model (U-Net) generally performed lower on this dataset compared to the other dataset which it was trained on (i.e., NIH).

**Table 4:** Pancreas segmentation performance comparison results obtained on MSD dataset

Models	DSC (%)	Precision (%)	Recall (%)	Jaccard index (%)
U-Net	64.36	69.26	66.78	53.40
Attention-UNet	64.80	**80.64**	80.60	**67.25**
UNet++	64.66	73.49	**88.07**	66.62
ResUNet	62.23	68.21	63.32	50.30
ResNet	64.09	67.93	67.34	52.75
**ResDAC-Net** **(*ours*)**	**69.15**	71.10	72.31	58.76

The best results are shown in bold

### Performance comparison with state-of-the-art models

Finally, we compared the segmentation performance of the proposed ResDAC model with that of state-of-the-art models as reported in the corresponding literature sources. The results are shown in Table 5. As can be seen, ResDAC outperforms all other models according to three (out of four) evaluation metrics, including the two main ones used for segmentation performance evaluation (i.e., DSC and Jaccard index). More specifically, the second-best performing model was left behind by 0.80 percentage points (w.r.t. ADAU-Net [53]) according to DSC, 3.88 percentage points (w.r.t. ADR-U-Net [54]) based on recall, and 1.32 percentage points (w.r.t. ADAU-Net [53]) according to Jaccard index.

**Table 5:** Pancreas segmentation performance comparison results according to literature

Models	DSC (%)	Precision (%)	Recall (%)	Jaccard index (%)
DAN [55]	83.31	84.09	83.30	71.76
ADR-U-Net [54]	83.03	-	84.60	-
MBU-Net [56]	82.87	**89.29**	77.37	70.97
ADAU-Net [53]	83.76	-	-	72.38
SE-PResUnet [57]	83.13	-	-	-
MCF [58]	75.02	-	-	61.27
SGU-Net [59]	80.72	-	-	-
MDAG-Net [22]	83.04	81.71	84.42	-
**ResDAC-Net (*ours*)**	**84.56**	81.69	**88.48**	**73.70**

The best results are shown in bold

## Conclusions and future directions

This paper has proposed a novel pancreas segmentation model, called ResDAC-Net, utilizing residual networks and asymmetric convolutions in its backbone, allowing it to significantly enhance the feature fitting and capture the pancreas’ position and shape edge features, thereby further improving its segmentation performance. The obtained experimental results demonstrate that ResDAC-Net achieves significant improvements in metrics, such as Dice similarity coefficient (DSC), precision, recall, and Jaccard index, compared to other existing models.

Although ResDAC-Net has achieved success in pancreas segmentation, there are still some unresolved issues. For instance, more effective preprocessing methods need to be used, such as targeted approaches to remove artifacts and noise, performing image registration, etc., which will contribute to the enhancement of the segmentation performance of the proposed model. Addressing distribution differences among different datasets is also a challenging problem worthy of in-depth research. By tackling these issues, more reliable organ segmentation can be achieved, which will have a positive impact in the field of medical imaging and clinical applications. In the future, we will strive to improve the generalization ability of the proposed model, design models for segmentation of other organs, and apply them to clinical experiments combined with hardware devices.
